# The relationship between expression of Toll-like receptor 4 in chronic hepatitis C patients and different stages of liver fibrosis 

**Published:** 2017

**Authors:** Salem Youssef Mohamed, Ehab Fawzy Mostafa, Amr Shaaban Hanafy, Hesham Atia, Ashraf Metwally, Ayman M. Marei

**Affiliations:** 1 *Internal Medicine Department, Hepatology Division, Zagazig University, Egypt *; 2 *Tropical Medicine Department, Faculty of Medicine, Zagazig University, Egypt*; 3 *Immunology Department, Zagazig University, Egypt*

**Keywords:** Toll-like Receptor 4, Chronic HCV patients, Liver fibrosis, Fibroscan, HCV RNA.

## Abstract

**Aim:**

The objective of this work is to find out whether there is a relation between the expression of TLR4 and fibrosis progression in chronic HCV patients.

**Background:**

Toll-like Receptor 4 (TLR4) is a pattern recognition receptor whose activation results in the production of several pro-inflammatory cytokines.

**Methods:**

Fifty patients with chronic HCV were included. They were divided into group A: 40 patients (F1-F4) and group B (control group) which included ten patients (F0) based on fibroscan value. All patients were exposed to clinical and laboratory evaluations preliminary to antiviral therapy, assessment of TLR4 mRNA by Real Time- PCR.

**Results:**

Twenty-eight males and 22 females with a mean age 28.9±6.1 years. The mean TLR4 expression is 11.2±7.4 folds, TLR4 expression in F0 group is 2.8±1.9, in F1 group 4.8±1.5, F2 group 10.2±2.5, F3 group 16.8±1.5 and in F4 21.3±3.6 folds (p<0.001). TLR4 showed a positive correlation with age, fibrosis stage, HCV RNA, serum transaminases, total bilirubin and prothrombin time, a negative correlation with platelet count and serum albumin. Fibrosis progression was independently associated with TLR4 expression (β=0. 648, P<0.0001), RNA (β= 0.160, P =0.001) and platelet count (β= -0.248, P = 0.004).

**Conclusion:**

The expression of TLR4 is highly correlated with the fibrosis progression; TLR4 may be a potential target for drugs to limit the progression of fibrosis.

## Introduction

 Liver fibrosis represents a liver response to injury with deposition of new collagen to repair the damage. By the time this process can result in cirrhosis of the liver with disruption of the functional units of the liver, thus affecting hepatic blood flow and function (1).

With the development of cirrhosis, portal hypertension and liver cell failure ensue, and the risk of liver cancer is highly increased (2). Hepatic stellate cells (HSC) are the main matrix-producing cells in the liver. When activated, they change to adopt the myofibroblast phenotype which can secrete collagen that can be remodeled through digestion of a matrix by matrix metalloproteinases (3).

Risk factors for developing liver fibrosis are chronic infection with hepatitis B or C virus, male gender, age over 50, compromised immune system, heavy alcohol consumption, fatty liver disease and insulin resistance (4).

Toll-like receptors (TLRs) are a class of proteins that play a fundamental role in the innate immunity. They are single, membrane-spanning, non-catalytic receptors usually expressed in sentinel cells, such as macrophages and dendritic cells. They recognize pathogen-associated molecular patterns that are expressed on infectious agents, and mediate the production of cytokines necessary for the development of active immunity. Once these microbes have breached physical barriers such as the skin or intestinal tract mucosa, they will be recognized by TLRs, which activate immune cell responses (5).

Despite the constant confrontation of hepatic TLR4 with gut-derived lipopolysaccharide (LPS), the healthy liver does not show signs of inflammation due to its low expression of TLR4 and its ability to modulate TLR-4 signaling. Nevertheless, there is accumulating evidence that altered LPS/TLR4 signaling is a key player in the pathogenesis of many chronic liver diseases (6).

TLR-4 has been shown to interact with lymphocyte antigen 96 (7), myeloid differentiation factor 88 (MyD88) (8) and toll interacting protein (TOLLIP) (9). Intracellular trafficking of TLR4 is dependent on the GTPase Rab-11a and knocks down of Rab-11a results in hampering TLR4 recruitment (10).

The healthy liver contains low mRNA levels of TLR4 and signaling molecules such as MyD88 in comparison to other organs; Kupffer cells (KC) because of the anatomical link between the liver and intestine are the first cell to encounter gut-derived toxins including LPS accordingly, KC expresses TLR4 and are responsive to LPS. Upon triggering, TLR4 signaling drives Kupffer cells to produce TNF-a, IL-1b, IL-6, IL-12, IL-18, and the anti-inflammatory cytokine IL-10 (11), the activated HSCs express TLR4 and CD14 and respond to LPS (12).

About 30% of patients chronically infected with HCV show signs of active hepatic inflammation and are at risk of developing fibrosis, cirrhosis, and hepatocellular carcinoma (HCC). There is accumulating evidence that LPS and TLR4 play a fundamental role in the pathogenesis of HCV infection (13).

HCV induces expression of TLR4 on the surface of B cells, leading to enhanced interferon (IFN)-b and IL-6 production and secretion, with increased tumor necrosis factor(TNF) receptor-associated factor and Interleukin receptor-associated kinase resulting in the activation of nuclear factor kappa beta(NF-κB) (14). 

TLR4 signaling in Kupffer cells and hepatocyte might constitute the link between chronic hepatic inflammation and HCC (15).

The aim of this work is to examine the expression of TLR4 in HCV-infected patients with different stages of fibrosis to identify the potential role of TLR4 in the induction of liver fibrosis. 

## Methods

The study was carried out at Zagazig University Hospitals, internal medicine department. From January 2014 to February 2015, out of 120 patients; 50 patients with chronic active HCV were included. The patients were divided into two groups: group A which included 40 patients (F1-F4) and group B (control group) which included ten patients (F0). Classification of the patients, according to fibrosis stage was based on fibroscan value.

**Exclusion criteria**

1. patients with hepatitis B co-infection,
2. Patients with body mass index over 28,
3. Patients with autoimmune diseases, bilharziasis, or abnormal thyroid function,
4. Pregnant women,
5. Patients who started antiviral therapy or taking medication affecting immunity as steroids or immunosuppressive agents.

**Laboratory analysis**

All patients underwent a 12-hour overnight fast before blood tests which included: 

A- Routine investigations are preliminary to combined therapy as Liver function tests, prothrombin time, prothrombin concentration (%), kidney function tests, complete Blood Count, fasting blood sugar, thyroid function tests, antinuclear antibodies, and Serum AFP. AST to platelet count ratio (APRI) was calculated also.

B- Real-time Quantitative PCR (COBAS Ampliprep/Taqman HCV monitor, with detection limit 15 IU/ml; Roche Diagnostic Systems). 

**Abdominal ultrasonography**

The patients were examined after 6 hours fast. Criteria of decompensated cirrhosis were documented. Measures of portal hypertension as Portal vein diameter more than 13mm, splenic bipolar diameter more than 130mm, and splenic vein diameter˃10mm were recorded (16).

**Fibroscan**

It was performed to measure liver stiffness; the number of shots is 10, success rate ≥ 60%, interquartile range≤ 25%. It measures liver stiffness in a volume like a cylinder 1 cm in diameter and 4 cm in length, between 25 and 65 mm underneath the skin surface. This volume is nearly 100 times bigger than a biopsy. Liver stiffness 2.5-7 kPs denotes (F0-1), 7-9.5 kPs (F2), 9.5-12.5 kPs (F3), >12.5 kPs denotes cirrhosis (17).

**TLR Expression**

*1. Collection of blood samples (within three days of the fibroscan)*

Two ml of venous blood was collected from each patient under the complete aseptic condition, left for 30-60 minutes for natural clotting then centrifuged at 3000 RPM for 10 minutes, and serum samples were separated into another set of tubes and kept frozen at -20 C until use for extraction of RNA.

*2. Extraction of RNA*

200 μl of the sample was transferred, and 200 μl of double distilled H2O was added. It was incubated for 15 minutes at 65°C in a thermomixer, then incubated for 10 minutes at 95°C in a thermomixer. Binding Solution (400 μl) was added. The sample was exposed to RTA Spin Filter.

*3. Real-time PCR*

Constitution of the expression levels of TLR-4 was performed by quantitative RT-PCR based on real-time PCR. Extracted total RNA using (Stratec, Germany) was reverse transcribed into cDNA by the manufacturer’s directions (Invitrogen, Carlsbad, CA).

TLR4 sense primer (5-GAACTGCAGGTGCTGGATTT-3), antisense primer (5-CTCTAGATTGGTCAGATTAGA-3). Probe (5_GTCCAGAAAAGGCTCCCAGGGCTAAAC-3). Both were used in association with green dye PCR master mix (Rovalab). The glyceraldehyde-3-phosphate dehydrogenase (GAPDH) level in each cDNA sample was also measured as a means of normalizing cytokine mRNA expression levels. The mRNA expression data are expressed as fold induction relative to the GAPDH level.

**Table 1:** Demographic and laboratory characteristics of the studied groups

P	F4	F3	F2	F1	F0	
<0.001	37.7±1.9	32.2±3.2	25.9±2.6	26.4±3.9	22.4±2.1	Age (Years)
<0.001	21.3±3.6	16.8±1.5	10.2±2.5	4.8±1.5	2.8±1.9	TLR4 expression(folds)
<0.001	130.4±6.8	805±6.1	561 ±4.1	375.4±1.9	319.6±2.2	RNA (KIU/ml)
<0.001	92.6±21.2	131±25.6	182±25.7	201±29.6	228.4±16.2	Platelet (cell/µl
<0.001	74.8±25	56.5±20	38.4±8.8	32.9±2.7	27.5±3.1	ALT (IU/L)
<0.001	65.1±21	53.6±16.5	32.4±10.1	26.4±2.6	21.8±3.6	AST (IU/L)
<0.001	1.8±0.7	1.1±0.47	0.5±14	0.31±.05	0.28±.04	APRI
<0.001	5.1±2.2	1.6±0.4	1.12±0.1	1.0±0.7	0.97±0.05	Total.Bilirubin (mg/dl)
<0.001	2.6±0.46	3.5±0.31	4.16±0.17	4.42±0.19	4.6±0.14	Albumin(gm/dl
<0.001	18.7±2.6	14.5±0.76	12.8±0.44	12.5±0.34	12.1±0.16	Prothrombin time

**Table 2:** Correlation of TLR4 expression with competing factors

Prothrombin time	Albumin	Bilirubin	AST	ALT	HCVRNA	Platelet count	Fibrosis stage	Sex	Age	
0.781	0.867	0.675	0.782	0.771	0.479	0.872	0.941	0.142	0.817	r
0.001	<0.001	<0.001	<0.001	<0.001	<0.001	<0.001	<0.001	0.325	<0.001	p

**Statistical analysis**

Data were statistically analyzed using SPSS version 21. Results were expressed as mean ± SD. Categorical variables were analyzed using the χ2 test, and continuous variables were analyzed using the Student’s t-test. Associations determined by correlation analysis were expressed as a Spearman’s correlation coefficient (r). Multivariable linear regression analysis was used to detect independent variables associated with hepatic fibrosis. P<0.05 was considered to be statistically significant. 

## Results

Fifty patients enrolled in this study; 28 males and 22 females with a mean age 28.9±6.1 years and a range of 20-40 years old. Laboratory investigations showed TLR4 expression with a range of 1-27 fold difference in average 11.2±7.4 folds; platelet count varied from 70-250x10³ cell/µl with a mean 167±54.6x10³ cell/µl. HCV RNA ranged from 40-2500 KIU/ml with a mean 673.04±5.8 KIU/ml, ALT mean value 44.4±23.6 IU/L, AST mean value 40.3±20.5 IU/L, total bilirubin mean value 2.0±1.9mg/dl, serum albumin mean value 3.9±0.8gm/dl.

Patients were divided into five subgroups according to results of fibroscan (table 1). A highly significant statistical difference was noted among patient subgroups as regards Age, TLR4 expression, ALT, AST, total bilirubin, prothrombin time being higher in an F4 subgroup, with serum albumin and platelet count being lower in them (p<0.001).

**Table 3:** Multivariate analysis to determine the independent variables related to fibrosis stage

	Beta coefficient ± SE	P
TLR4 expression	0.648	<0.001
HCV RNA	0.160	0.001
Platelet count	-0.248	0.004

**Figure 1:**
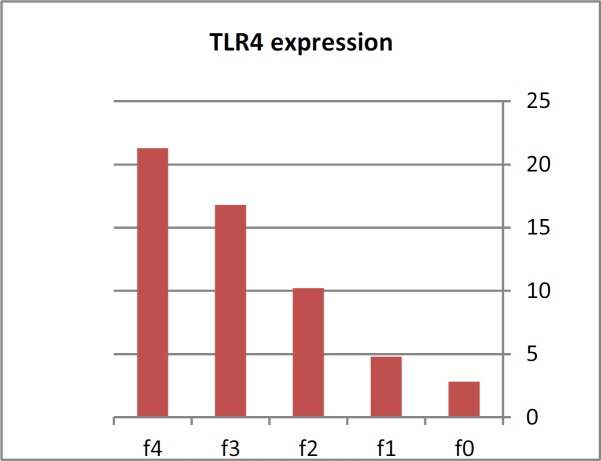
TLR4 expression in different stages of liver fibrosis.

**Figure 2.**
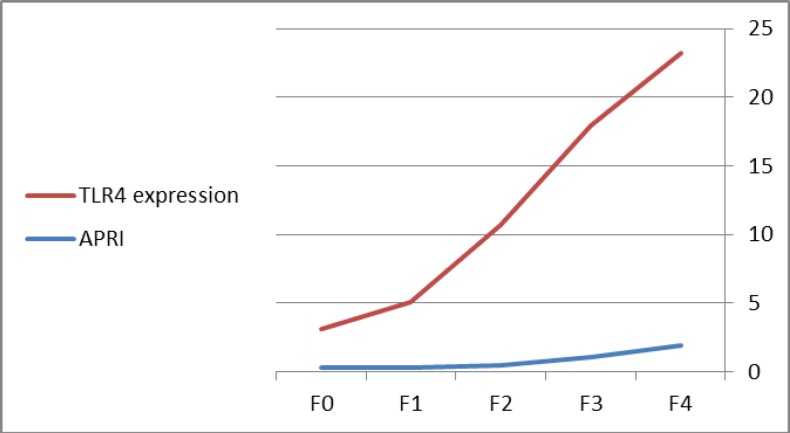
TLR4 was correlated with APRI value as their values were increased with progression of fibrosis stage

Spearman rank correlation was done to show factors closely correlated with the TLR4 expression and revealed that high positive correlation with age, fibrosis stage, HCV RNA, ALT, AST, total bilirubin and prothrombin time, a strong negative correlation with platelet count and serum albumin as shown in Table 2. Multivariate linear regression analysis to determine variables independently associated with stage of fibrosis, which revealed that TLR4 expression (β=0.648, p<0.001), HCV RNA (β= 0.160, p=0.001) and platelet count (β= -0.248, p=0.004) were mostly associated with progression of fibrosis as shown in Table 3. TLR4 expression was enhanced with progression of fibrosis stage with male predominance as shown in fig 1. TLR4 was correlated with Aspartate transaminase to platelet ratio index (APRI) as their values were increased with progression of fibrosis stage as shown in fig 2.

## Discussion

TLR4 is a transmembrane receptor recognizing lipopolysaccharide as its central legend. Activation of TLR4 causes inflammation by promoting the secretion of inflammatory cytokines (18).

Knowing the mechanism of fibrosis and the molecules and receptors implicated in its progression may be the next wave in the management of complications of chronic HCV infection. TLR4 signaling was considered to initiate fibrogenesis by pro-inflammatory and pro-fibrogenic cytokines from Kupffer cells, which then activate HSC (19).

TLR4 signaling drives Kupffer cells to produce TNF-a, IL-1b, IL-6, IL-12, IL-18, and the anti-inflammatory cytokine IL-10 (11). TLR4 activation leads to boosting of TGF- signaling with subsequent hepatic fibrosis through down-regulation of the transforming growth factor (TGF)-beta (20). The objective of this work is to find out whether there is a relation between the expression of TLR4 and fibrosis progression in chronic HCV patients.

This study was carried out on fifty patients. The patients were divided into two groups: the first group (study group) forty patients (F1-F4) and the second group (control group) ten patients (F0). The results revealed that liver fibrosis progression has a positive correlation with age, TLR4 expression, viral load, ALT, AST, total bilirubin and PT, and negative correlation with platelet count and serum albumin. TLR4 expression, platelet count, and viral load were more independent variables associated with fibrosis progression.

The expression of TLR4 correlates with the progression of fibrosis and this in agreement with a study made by Machida *et al*. (21) which showed that HCV, through the action of its NS5A protein, induces expression of TLR4, leading to enhanced IFN-beta and IL-6 production and secretion. So, HCV infection causes an inflammatory response and antiviral state at the same time through the effects on TLR4 expression. 

Seki *et al.* (20) showed that an active contribution of LPS- TLR4 interaction in the development of liver fibrosis. Systemic plasma LPS levels were significantly elevated in mouse models of experimental liver fibrosis.

This study revealed that HCV RNA viral load correlated with the progression of fibrosis and is in agreement with a study made by Fanning et al. who identified that HCV RNA load was associated with hepatic inflammation (22).

It was shown that a highly significant positive correlation involved TLR4 expression, the progression of liver fibrosis, age, RNA, transaminases, total bilirubin and prothrombin time, and a highly significant negative correlation with platelet count and serum albumin**.**

A study made by Vespasiani-Gentilucci *et al*. found a significant correlation between TLR4 expression by hepatic progenitor cells and biliary epithelial cells, and grade of inflammation, mainly interface activity, activation of portal/septal myofibroblasts, and liver fibrosis (23).

Several single nucleotide polymorphisms have identified that predict reduced TLR4 responsiveness and confer a significantly reduced risk for fibrosis progression (24). 

The TLR4 expression is correlated with the progression of fibrosis and its blockage may give new hope to decrease the progression of fibrosis, since a significant achievement in the drug therapy of chronic HCV had been made. The inevitable progression of fibrosis even after viral clearance remains a challenge and should enhance the next wave of studies to fight it. Finally, we recommend more studies with a greater sample size to clarify this issue.

## Conflict of interests

The authors declare that they have no conflict of interest.
